# A Fibrosis Biomarker Early Predicts Cardiotoxicity Due to Anthracycline-Based Breast Cancer Chemotherapy

**DOI:** 10.3390/cancers14122941

**Published:** 2022-06-14

**Authors:** Ana de la Fuente, Marta Santisteban, Josep Lupón, José Manuel Aramendía, Agnes Díaz, Ana Santaballa, Amparo Hernándiz, Pilar Sepúlveda, Germán Cediel, Begoña López, José María López Picazo, Manuel M. Mazo, Gregorio Rábago, Juan José Gavira, Ignacio García-Bolao, Javier Díez, Arantxa González, Antoni Bayés-Genís, Susana Ravassa

**Affiliations:** 1 Department of Cardiology and Cardiac Surgery, Clínica Universidad de Navarra, 31008 Pamplona, Spain; adelafuente.1@unav.es (A.d.l.F.); adiazdo@unav.es (A.D.); grabago@unav.es (G.R.); jjgavira@unav.es (J.J.G.); igarciab@unav.es (I.G.-B.); jadimar@unav.es (J.D.); 2 Breast Cancer Unit, Medical Oncology Department, Clínica Universidad de Navarra, 31008 Pamplona, Spain; msantisteb@unav.es (M.S.); jmaramen@unav.es (J.M.A.); jlpicazo@unav.es (J.M.L.P.); 3 IdiSNA, Navarra Institute for Health Research, 31008 Pamplona, Spain; blopez@unav.es (B.L.); mmazoveg@unav.es (M.M.M.); amiqueo@unav.es (A.G.); 4 CIBERCV, Carlos III Institute of Health, 28029 Madrid, Spain; jlupon.germanstrias@gencat.cat (J.L.); pilar.sepulveda.sanchis@gmail.com (P.S.); abayesgenis@gmail.com (A.B.-G.); 5 Servei de Cardiologia i Unitat d’Insuficiència Cardíaca, Hospital Universitari Germans Trias i Pujol, 08916 Badalona, Spain; gecediel@yahoo.com; 6 Department of Medicine, Universitat Autònoma de Barcelona, 08193 Barcelona, Spain; 7 Clinical and Translational Research in Cancer, Instituto de Investigación Sanitaria La Fe, 46026 Valencia, Spain; santaballa_ana@gva.es; 8 Regenerative Medicine and Heart, Instituto de Investigación Sanitaria La Fe, 46026 Valencia, Spain; hernandiz_amp@gva.es; 9 Program of Cardiovascular Diseases, Cima Universidad de Navarra, 31008 Pamplona, Spain; 10 Regenerative Medicine Program, Cima Universidad de Navarra and Hematology and Cell Therapy Area, Clínica Universidad de Navarra, 31008 Pamplona, Spain; 11 ICREC Research Program, Germans Trias i Pujol Health Science Research Institute, 08916 Badalona, Spain

**Keywords:** anthracycline-based chemotherapy, myocardial fibrosis, biomarkers, global longitudinal strain, cardiotoxicity

## Abstract

**Simple Summary:**

Left ventricular dysfunction (LVD) induced by anthracycline-based cancer chemotherapy (ACC) is becoming an urgent healthcare concern. Myocardial fibrosis (MF) may contribute to LVD after ACC. We show that elevated circulating levels of procollagen type I C-terminal propeptide (PICP, biomarker of MF) are associated with early subclinical LVD and predict later development of cardiotoxicity in patients treated with ACC. In addition, an association between PICP and LVD in patients with ACC-induced heart failure is observed. These results provide novel insights into MF as a mechanism underlying LVD after ACC, with PICP emerging as a promising tool to monitor cardiotoxicity in patients treated with ACC.

**Abstract:**

Anthracycline-based cancer chemotherapy (ACC) causes myocardial fibrosis, a lesion contributing to left ventricular dysfunction (LVD). We investigated whether the procollagen-derived type-I C-terminal-propeptide (PICP): (1) associates with subclinical LVD (sLVD) at 3-months after ACC (3m-post-ACC); (2) predicts cardiotoxicity 1-year after ACC (12m-post-ACC) in breast cancer patients (BC-patients); and (3) associates with LVD in ACC-induced heart failure patients (ACC-HF-patients). Echocardiography, serum PICP and biomarkers of cardiomyocyte damage were assessed in two independent cohorts of BC-patients: CUN (*n* = 87) at baseline, post-ACC, and 3m and 12m (*n* = 65)-post-ACC; and HULAFE (*n* = 70) at baseline, 3m and 12m-post-ACC. Thirty-seven ACC-HF-patients were also studied. Global longitudinal strain (GLS)-based sLVD (3m-post-ACC) and LV ejection fraction (LVEF)-based cardiotoxicity (12m-post-ACC) were defined according to guidelines. BC-patients: all biomarkers increased at 3m-post-ACC versus baseline. PICP was particularly increased in patients with sLVD (interaction-*p* < 0.001) and was associated with GLS (*p* < 0.001). PICP increase at 3m-post-ACC predicted cardiotoxicity at 12m-post-ACC (odds-ratio ≥ 2.95 per doubling PICP, *p* ≤ 0.025) in both BC-cohorts, adding prognostic value to the early assessment of GLS and LVEF. ACC-HF-patients: PICP was inversely associated with LVEF (*p* = 0.004). In ACC-treated BC-patients, an early increase in PICP is associated with early sLVD and predicts cardiotoxicity 1 year after ACC. PICP is also associated with LVD in ACC-HF-patients.

## 1. Introduction

In patients with breast cancer (BC), highly-effective oncologic drugs are associated with side effects, cardiotoxicity among them, which limits treatment options and contributes to morbidity and mortality [1]. Therefore, there is a critical need to understand the pathophysiological mechanisms activated in the myocardium of patients undergoing BC chemotherapy before irreversible cardiac damage occurs [2]. In addition, new diagnostic and prognostic biomarkers of cardiotoxicity are needed in these patients, especially in those in whom the presence of left-sided implants may affect the assessment of myocardial structure and function by cardiac imaging techniques [3,4].

Traditionally, cardiotoxicity due to BC treatment, particularly anthracycline-based cancer chemotherapy (ACC), has been attributed to cardiomyocyte damage and death, with amino-terminal pro-brain natriuretic peptide (NT-proBNP) and high-sensitivity troponins (hs-Tn) as the most commonly proposed biomarkers to detect cardiotoxicity [2,5,6]. However, it has been suggested that myocardial fibrosis is an additional important mechanism contributing to left ventricular dysfunction (LVD) and adverse clinical evolution in ACC-treated patients [5,7]. As cumulative evidence suggests that the detrimental impact of myocardial fibrosis on LV function is related to both an excess in collagen type I deposition and increased fibers cross-linking [8], these characteristics of the collagen fibers should be evaluated in the myocardium of ACC-treated patients. In this regard, some circulating peptides with histologically-proven association with myocardial fibrosis have been described. On the one hand, the serum procollagen type I C-terminal propeptide (PICP), released during the conversion of procollagen type I into fibril-forming mature collagen type I, is associated with LV myocardial collagen type I deposition [9,10]. On the other hand, the ratio of serum collagen type I C-terminal telopeptide to serum matrix metalloproteinase-1 (CITP:MMP-1) is inversely correlated with LV myocardial collagen cross-linking, as the higher is the cross-linking among collagen type I fibrils, the lower will be the cleavage of CITP by MMP-1 during the process of fiber degradation [11]. 

Therefore, we have investigated whether ACC induces biomarker-assessed myocardial fibrosis and whether these biomarkers are associated with early subclinical LVD and predict LV ejection fraction (LVEF)-based cardiotoxicity in BC patients treated with ACC, either with epirubicin (Clínica Universidad de Navarra [CUN]) or with doxorubicin (Hospital Universitari i Politecnic La Fe [HULAFE]). In addition, we have studied whether biomarkers of myocardial fibrosis are associated with LVD in ACC-induced heart failure (ACC-HF). To address these questions, we have analyzed changes in the levels of serum PICP and CITP:MMP-1, and of biomarkers associated with myocardial injury-dysfunction (hs-TnT and NT-proBNP) in BC patients before ACC and at post-treatment visits up to 12 months after ACC completion. Moreover, we have validated our findings in an independent cohort of ACC-treated BC patients. Finally, we have analyzed PICP levels in ACC-HF patients in whom LV function has been evaluated at baseline and after 12 months of guideline-guided treatment.

## 2. Materials and Methods

All procedures performed in studies involving human participants followed the ethical standards of the Clinical Investigation Ethics Committees of the University of Navarra, the Hospital Universitari i Politecnic La Fe, and the Hospital Universitari Germans Trias i Pujol. The study conformed to the principles of the Helsinki Declaration of 1975, as revised in 1983.

### 2.1. Patients with BC (CUN Cohort)

Samples were provided by the Biobank of the University of Navarra and were processed following standard operating procedures approved by the Clinical Investigation Ethics Committee of the University of Navarra.

Patients with treatment-naive primary BC were prospectively recruited from 2018 to 2020 in the Breast Cancer Unit at the Department of Medical Oncology at the Clínica Universidad de Navarra (CUN). Patients were at least 18 years old and had a diagnosis of BC stage I-III with an indication to receive adjuvant or neoadjuvant chemotherapy. The standard chemotherapy protocol consisted of epirubicin 90 mg/m^2^ and cyclophosphamide 600 mg/m^2^ cycled every two (neoadjuvant) or three (adjuvant) weeks for 4 cycles, followed by 12 weekly cycles of paclitaxel (80 mg/m^2^) or 4 cycles of docetaxel (100 mg/m^2^) every 21 days. Patients with Her-2 positive invasive breast cancer received trastuzumab with/without pertuzumab simultaneously with taxanes and continued later until completing one year of treatment. The patients received complementary radiotherapy according to standard protocols. Exclusion criteria included a history of congenital heart disease, coronary heart disease, heart valve disease, heart failure, or LVEF < 50%.

#### 2.1.1. Study Protocol

**Figure 1:**
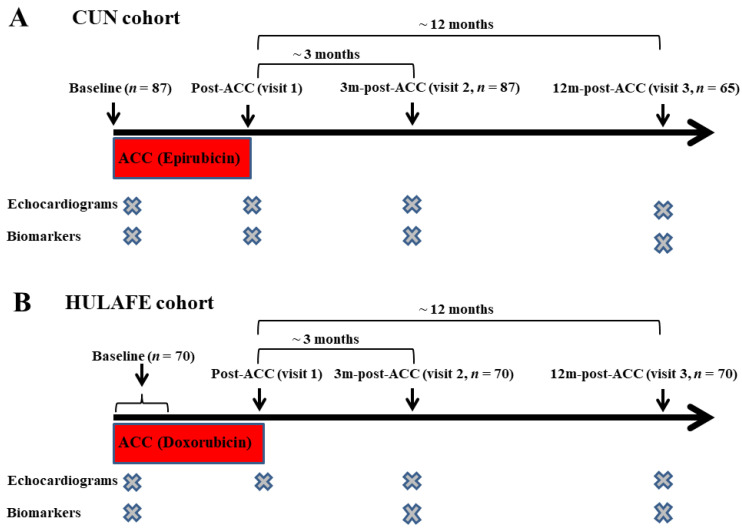
Study protocol in patients with breast cancer. The diagrams depict the study protocols in the CUN (**A**) and HULAFE (**B**) cohorts. Patients with breast cancer were studied before (baseline) and after ACC (post-ACC), and at 3 and 12 months post-ACC.

Serum samples and echocardiographic parameters were analyzed before chemotherapy (baseline), after epirubicin/cyclophosphamide treatment (post-ACC), and approximately at 3 months (median: 2.9 [interquartile range (IQR): 2.3–3.4]) (3m-post-ACC) and 12 months (median: 12.2 [interquartile range (IQR): 10.7–14.2) (12m-post-ACC) after completion of ACC therapy (Figure 1A).

Three-dimensional (3D) echocardiographic studies were performed on all patients using a Philips Epic 7 echocardiography system with an X5-1 transducer. The echocardiography studies’ data were stored digitally in the Dicom server. 3D LV volumes and LVEF were measured using Q-Lab software. LV longitudinal myocardial strain and automated function imaging were assessed by an experienced investigator using the automated cardiac motion quantification (aCMQ) on the installed Q-Lab software. For the 2D Speckle-tracking analysis, the software automatically traced the myocardial motion on the previously acquired images of four-chamber, two-chamber and three-chamber standard apical views. The operator adjusted manually the myocardial limits if automated tracking was considered inaccurate. Global longitudinal strain (GLS) was obtained from an average of regional longitudinal strain measured from a 17 myocardial segments model [12]. All measurements were made by a single observer who was blinded to all clinical and biomarker data. The intra-observer coefficients of variation for GLS and LVEF were 3.1% and 4.4%, respectively.

#### 2.1.2. Study Endpoints

Early ACC-induced subclinical LVD at 3 months-post-ACC was defined as a relative reduction in global longitudinal strain (GLS, as assessed by speckle-tracking analysis) >1 5% versus baseline [13], and ACC-induced cardiotoxicity at 12 months post-ACC was defined as a decrease in LVEF > 15% or a decrease of LVEF > 10% to an absolute value < 55% versus baseline [13].

### 2.2. Patients with BC (HULAFE Cohort)

Samples from 70 patients with BC recruited from 2013 to 2015 were provided by the Biobank of the Hospital Universitari i Politecnic La Fe (HULAFE) and were processed following standard operating procedures approved by the Clinical Investigation Ethics Committee of the Hospital Universitari i Politecnic La Fe. 

These patients underwent two different ACC protocols:

-ACC1: Patients were treated with docetaxel 75 mg/m^2^, doxorubicin (adriamycin) 50 mg/m^2^ and cyclophosphamide 500 mg/m^2^, cycled every 21 days for 6 cycles;

-ACC2: Patients were treated with doxorubicin (adriamycin) 60 mg/m^2^ and cyclophosphamide 600 mg/m^2^ cycled every 21 days for 4 cycles, and then paclitaxel (80 mg/m^2^) or docetaxel (100 mg/m^2^) weekly for 12 weeks or cycled every 21 days for 4 cycles, respectively. Of these patients, those with Her-2-positive invasive BC were further treated with trastuzumab (herceptin) 600 mg cycled every 21 days for 12 cycles.

#### 2.2.1. Study Protocol

Serum samples and echocardiographic parameters were analyzed before or during ACC (baseline) and at 3m- (median: 3.1 [IQR: 1.6–4.1]) and 12m-post-ACC (median: 12.6 [interquartile range (IQR): 11.4–15.4]). LVEF and GLS measurements were also available post-ACC (Figure 1B).

Conventional 2D echocardiography was performed following the American Society of Echocardiography guidelines using the iE33 scanner (Philips Medical Systems, Andover, MA) with transthoracic S5-1 and X5-1 broadband transducers (frequency = 1–5 MHz). Cross-sectional images were recorded from the apex, and end-diastolic and end-systolic areas and LV lengths were measured from the apical four-chamber (A4C) and two-chamber (A2C) views (using the modified biplane Simpson’s method) for the calculation of LVEF. All tracings were made at a centralized reading center by a single observer who was blinded to all other clinical and biomarker data. GLS was determined by speckle tracking with the Automated Cardiac Motion Quantification (aCMQ, QLAB 9; Philips Medical Systems- Andover, MA).

#### 2.2.2. Study Endpoints

Cardiotoxicity at 12 months post-ACC was defined as previously explained.

### 2.3. Patients with ACC-Induced HF

These patients were part of a larger HF cohort previously described [14]. In summary, all patients were ambulatory HF patients treated at a multidisciplinary unit from the Division of Cardiology at the Hospital Universitari Germans Trias i Pujol (Badalona, Spain). The inclusion criteria for the current study were ACC-induced HF and the availability of baseline echocardiogram and biomarker measurements. 

Thirty-seven patients with ACC-HF were included, of whom 24 (64.9%) had been diagnosed with BC. Anthracyclines (median [IQR] cumulative doses for epirubicin and doxorubicin: 540 (450–630) and 300 (278–413) mg/m^2^, respectively) were given a median (IQR) of 7.0 (1.7–12.6) years ago, and the presence of cardiovascular comorbidities was further examined to exclude causes other than ACC therapy as the main driver of symptomatic HF. LVEF measurements after 1-year guideline-guided therapy were available in 33 patients.

### 2.4. Circulating Biomarkers

Blood samples were taken from the antecubital vein at each visit, kept on ice for 1–2 h maximum before processing, aliquoted, and kept at −80 °C until measured. Additionally, samples were batch-analyzed to minimize differences due to sample processing and storage. All biomarkers were measured in serum. NT-proBNP and hs-TnT were measured using an electrochemiluminescence immunoassay (Roche Diagnostics, Meylan, France). The inter-and intra-assay coefficients of variation for both biomarkers were less than 10%. The lower limits of detection for NT-proBNP and hs-TnT were 5 pg/mL and 3 ng/L, respectively. CITP was measured by radio-immunoassay (Orion Diagnostica, Espoo, Finland). The inter-assay and intra-assay coefficients of variation were 10.2% and 9.3%, respectively. The lower limit of detection was 0.6 µg/L. MMP-1 was measured by an alphaLISA (Perkin Elmer, Waltham, MA, USA). The inter-assay and intra-assay coefficients of variation were 10.7% and 1.6%, respectively. The lower limit of detection was 82.6 pg/mL. CITP and MMP-1 values were expressed in molarity and their ratio was calculated in each patient as previously reported [11]. PICP was measured using the EIA MicroVue CICP (Quidel Corporation, San Diego, CA, USA). The inter-assay and intra-assay coefficients of variation were 9.1% and 4.1%, respectively. The lower limit of detection was 0.2 ng/mL.

### 2.5. Statistical Analysis

Non-normally distributed variables were examined after logarithmic transformation. Given the skewed distributions, all biomarkers were log2 transformed, allowing comparison of the associations as each 1-unit increase is equivalent to a doubling in biomarker levels. Differences between two groups of subjects were tested by Student’s t-test for unpaired data once normality was demonstrated. Otherwise, a nonparametric test (Mann-Whitney U test) was used. Categorical variables were analyzed by using the chi-squared test or Fisher’s exact test when necessary. Mixed-effects regression modeling was used for longitudinal association analyses. Individual linear mixed-effects models with a random intercept to account for intraparticipant correlation of repeated measures were used to describe changes across visits. In BC patients, the presence or absence of subclinical LVD or cardiotoxicity (at 3- or 12-months post-ACC, respectively), the visit, their interaction term, and baseline values of the dependent variable were added as fixed effects to examine whether the development of LVD was accompanied by greater changes in the parameters of interest as compared with its absence over time. Visit as a random slope was allowed if the model was significantly better fitted compared to a model with only a random intercept. The variance component structure was specified according to the best fit model. The likelihood ratio test and Akaike information criterion were used to select the model with the best fit. To examine the independent association between PICP and GLS as the dependent variable, confounding variables with *p* values ≤ 15 in univariate analyses, including baseline GLS values, were considered in the multivariable analysis. Multicollinearity was defined as variance inflation factor (VIF) > 5. The heteroscedasticity and normality of the residuals were checked graphically. 

Partly conditional logistic models were employed to determine longitudinal associations between repeated assessments of changes in biomarkers from baseline to 3m-post-ACC and the risk of cardiotoxicity at 12 months post-ACC [15,16]. This approach provides a flexible framework that allows for the estimation of the best unbiased linear predictors for each biomarker trajectory across time using mixed-effect models. All analyses were adjusted for baseline biomarker values (model 1). In addition, analyses were adjusted for age and ACC cumulative dose or treatment protocol (model 2) as variables associated with subclinical LVD in CUN patients that also showed differences between HULAFE patients with and without cardiotoxicity. Moreover, adjustment for the presence or absence of cardiovascular comorbidities was performed (model 3). Finally, because renal clearance of biomarkers may be important, adjustments were also performed for eGFR (model 4). Model calibration was assessed using the Hosmer–Lemeshow goodness-of-fit statistics. The incremental predictive value was determined by c-statistic, or area under the receiver operating characteristic curves (AUC), integrated discrimination (IDI), and net reclassification (NRI) improvements. The variances for the NRI estimates were calculated using bootstrapping (1000 resamples).

The Benjamini and Hochberg multiple test correction (false discovery rate of 5%) was applied to all biomarker analyses.

Mixed-effect analyses were performed with the “mixed” command within STATA. Partly conditional logistic models and AUCs were computed with the “partlyconditional” and “pROC” R packages, respectively. IDI and NRI analyses were performed with the “PredictABEL” and “nricens” R packages, respectively. Values are expressed as mean±SD or median (interquartile range), and categorical variables as numbers and percentages. Statistical significance was set as a 2-sided *p* of 0.05. The statistical analyses were performed by using the STATA (13.0 version) and RStudio (2022.02.0 version) software.

## 3. Results

### 3.1. Serum Biomarkers and ACC-Induced Subclinical LVD at 3 Months-Post-ACC in BC Patients (CUN Cohort)

One hundred patients with treatment-naive primary BC were prospectively recruited from 2018 to 2020. A total of 87 patients with planned treatment with epirubicin/cyclophosphamide were eligible for inclusion (mean age 50.8 years, range 31–81 years). Reasons for exclusion included loss to follow-up (*n* = 8), incomplete baseline and follow-up data (*n* = 3), and unwillingness or inability to provide informed consent (*n* = 2). The baseline characteristics of these patients are shown in Table 1.

Longitudinal analyses showed that NT-proBNP and hs-TnT increased post-ACC and at 3 months post-ACC compared with baseline. In addition, GLS showed a relative reduction and PICP increased at 3 months post-ACC compared with post-ACC and baseline (Supplementary Table S1).

**Figure 2:**
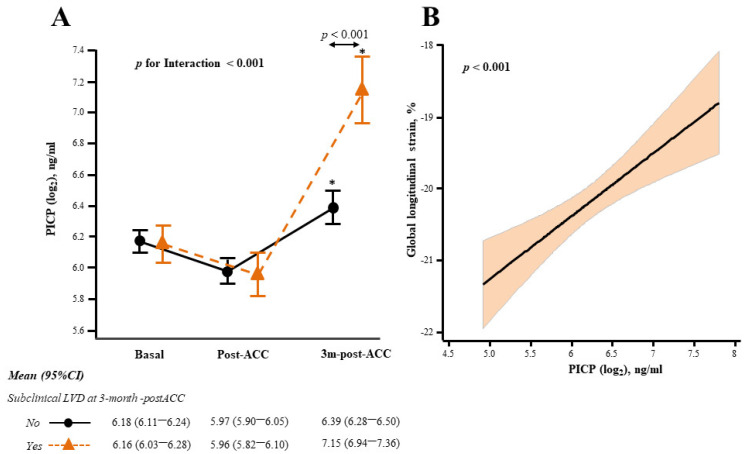
PICP and early cardiotoxicity in BC patients treated with ACC. (**A**) PICP circulating levels in patients with (orange triangle) or without (black circle) subclinical LVD 3 months after completion of ACC (3m-post-ACC). Data are expressed as the estimated marginal means and 95% confidence interval (CI) after linear mixed regression (LMR) analysis with PICP as the dependent variable, and subclinical LVD (yes/no), visit, their interaction term, estimated glomerular filtration rate, and PICP baseline values as fixed effects. * *p* < 0.001 vs baseline and post-ACC. (**B**) Association of PICP with global longitudinal strain (GLS) (solid black line) and 95% confidence intervals (color shade) throughout the treatment up to 3m-post-ACC after multivariable LMR with adjustment as explained in the main text. PICP, procollagen type-I C-terminal propeptide; BC, breast cancer; ACC, anthracycline-based cancer chemotherapy; LVD, left ventricular dysfunction.

Eighteen patients (23.0%) exhibited subclinical LVD at 3 months post-ACC. No clinically relevant differences were observed at baseline between these patients and those with unchanged GLS values (Table 1). Of interest, interaction analyses showed that patients with early subclinical LVD exhibited lower LVEF (3D) and higher PICP levels than patients with normal LV function (*p* for interaction < 0.001) (Supplementary Table S2). The interaction observed for PICP was confirmed after adjustment for the estimated glomerular filtration rate (eGFR) (Figure 2A). No significant interactions were found for the remaining biomarkers (Supplementary Table S2).

Univariable linear analyses showed that NT-proBNP, hs-TnT and PICP were directly associated with a relative reduction of GLS (Table 2). Multivariable adjustment revealed that a progressive increment in PICP levels predicted the relative reduction of GLS independently of age, body mass index, epirubicin cumulative dose, eGFR, levels of aspartate aminotransferase, NT-proBNP, hs-TnT, and GLS at baseline (Table 2, Figure 2B).

### 3.2. Serum Biomarkers and Prognosis of ACC-Induced Cardiotoxicity at 12 Months Post-ACC in BC Patients (CUN and HULAFE Cohorts)

Four patients who presented cardiotoxicity, as assessed by LVEF, at 3 months post-ACC, and who were to receive cardioprotective therapies, were excluded from this analysis since LVD was already present and the prognostic value of the biomarkers could not be assessed. Of interest, these patients had remarkably higher PICP levels at 3 months post-ACC (213 [IQR: 207–222] ng/mL) than the patients remaining in the study (85.1 [IQR: 69.8–115] ng/mL). In addition, eight patients left the study prematurely, and 10 patients had incomplete data. In total, echocardiographic parameters of systolic function (i.e., GLS, LVEF), and the biomarkers NT-proBNP, hs-TnT, and PICP, were further examined in 65 CUN patients 12 months after completion of ACC therapy. These parameters were also evaluated in the 70 HULAFE patients.

**Figure 3:**
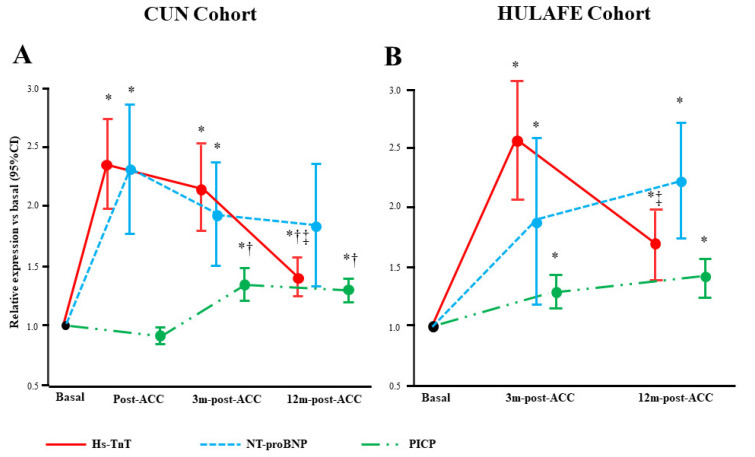
Biomarker levels at different visits until 12 months post-ACC in BC patients. Data are biomarker fold change levels relative to baseline values in the CUN (**A**) and HULAFE (**B**) cohorts, expressed as the estimated marginal means and 95% confidence interval (CI) after linear mixed regression analyses. * *p* < 0.01 vs. baseline, † *p* < 0.01 vs completion of ACC (post-ACC), ‡ *p* < 0.01 vs. 3 months post-ACC. BC, breast cancer; ACC, anthracycline-based cancer chemotherapy; NT-proBNP, amino-terminal pro-brain natriuretic peptide; hs-TnT, high-sensitivity troponin T; PICP, procollagen type-I C-terminal propeptide.

Compared with baseline, the levels of hs-TnT and PICP were elevated throughout all visits in both CUN and HULAFE patients ( Supplementary Table S3, Figure 3). Whereas this was also the case for NT-proBNP in the HULAFE patients, this biomarker was slightly decreased at the end of the study in the CUN patients ( Supplementary Table S3, Figure 3)

Ten (15.4%) CUN patients and eleven (15.7%) HULAFE patients exhibited cardiotoxicity at 12 months post-ACC. No clinically relevant differences were observed at baseline in CUN patients classified according to the absence or presence of cardiotoxicity (Table 3, Supplementary Table S4). The HULAFE patients who exhibited cardiotoxicity were younger, and more frequently treated with the ACC1 protocol (Table 3).

**Figure 4:**
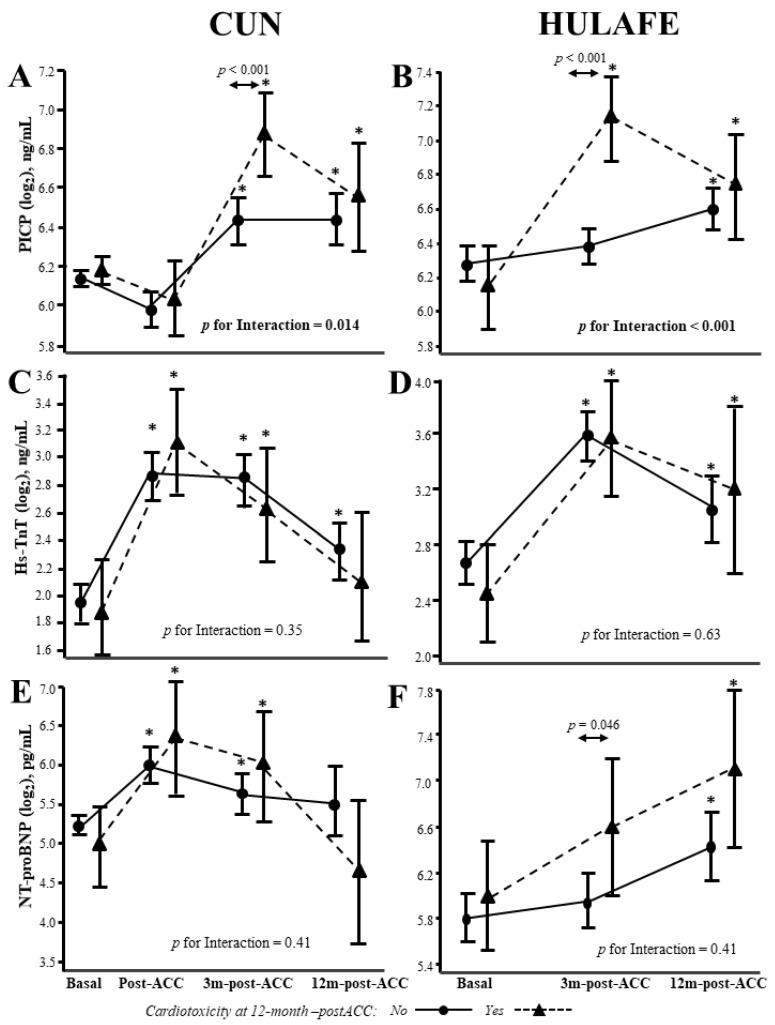
Biomarker levels at different visits in BC patients with presence or absence of cardiotoxicity at 12 months post-ACC. PICP (**A**,**B**), hs-TnT (**C**,**D**) and NT-proBNP (**E**,**F**) circulating levels in patients from the CUN and HULAFE cohorts, respectively, according to the presence (triangle) or absence (circle) of cardiotoxicity at 12 months after completion of ACC (12m-post-ACC). Data are expressed as the estimated marginal means and 95% confidence interval (CI) after linear mixed regression analyses with the biomarker as the dependent variable, and cardiotoxicity (yes/no), visit, their interaction term, and biomarker baseline values as fixed effects. * *p* < 0.05 vs baseline. BC, breast cancer; ACC, anthracycline-based cancer chemotherapy; NT-proBNP, amino-terminal pro-brain natriuretic peptide; hs-TnT, high-sensitivity troponin T; PICP, procollagen type-I C-terminal propeptide.

Of interest, interaction analyses in both cohorts showed that patients with cardiotoxicity at 12 months post-ACC exhibited higher PICP levels at 3 months post-ACC than patients without cardiotoxicity (Figure 4A,B, Table 4). No interactions were found for hs-TnT and NT-proBNP (Figure 4C–F, Table 4).

**Figure 5:**
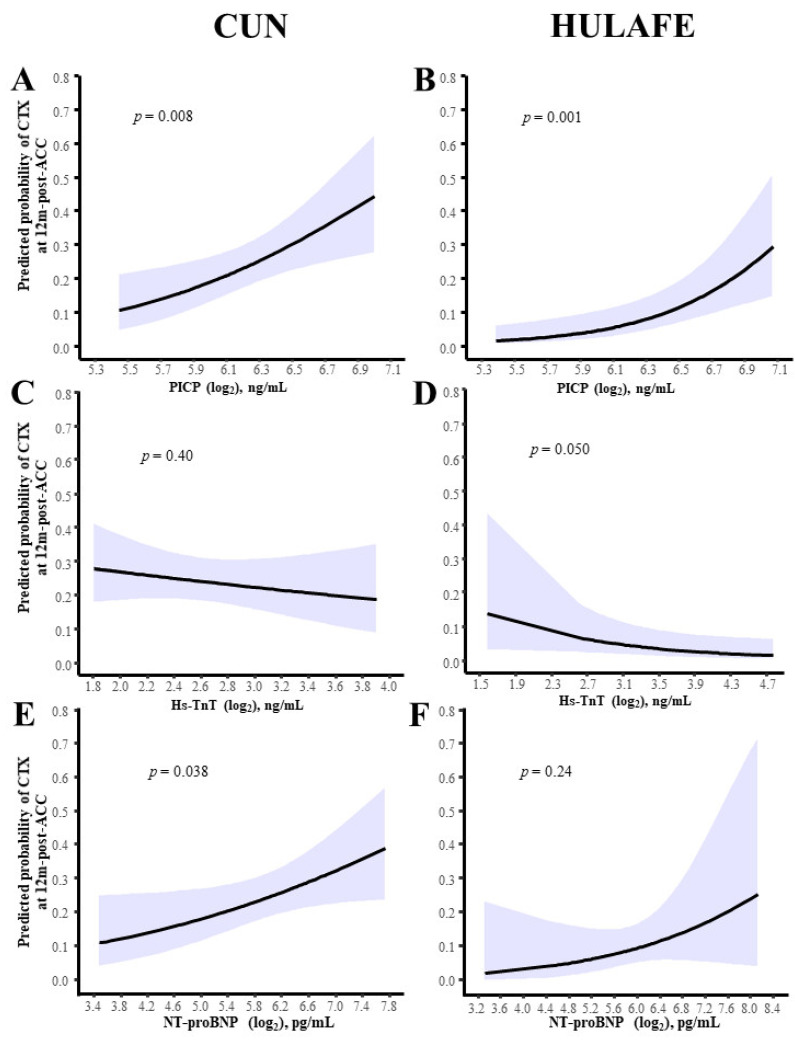
Biomarker changes from baseline to 3m-post-ACC and probability of cardiotoxicity at 12m-post-ACC in BC patients. Predicted probability of cardiotoxicity (solid black line) and 95% confidence intervals (color shade) at 12 months after completion of ACC as a function of PICP (**A**,**B**), hs-TnT (**C**,**D**) and NT-proBNP (**E**,**F**) variations (log2) from baseline to 3m-post-ACC, in the CUN and HULAFE cohorts, after partly conditional logistic regression analyses with adjustment for the respective biomarker baseline values. BC, breast cancer; ACC, anthracycline-based cancer chemotherapy; NT-proBNP, amino-terminal pro-brain natriuretic peptide; hs-TnT, high-sensitivity troponin T; PICP, procollagen type-I C-terminal propeptide.

Partly conditional logistic regression showed that an increment in PICP from baseline to 3 months post-ACC was associated with a higher probability of developing cardiotoxicity at 12 months post-ACC in both cohorts (Figure 5A,B), in all models considered (Table 5), whereas no independent associations were found for hs-TnT and NT-proBNP (Figure 5C–F, Table 5). 

The addition of PICP to partly conditional logistic models including GLS or LVEF changes from baseline to 3m-post-ACC improved discrimination and reclassification of CUN and HULAFE patients at risk of cardiotoxicity 12 months post-ACC (Table 6).

### 3.3. Serum Biomarkers and LVD in ACC-HF Patients

In a cohort of patients with ACC-induced HF, doublings of NT-proBNP and PICP were associated with lower LVEF (−2.80% [95%CI: −4.44 to −1.17], r = −0.510, *p* = 0.001 and −10.5% [95%CI: −17.4 to −3.66], r = −0.469, *p* = 0.004, respectively). PICP inverse association with LVEF remained significant after controlling for age and sex (−10.8% [95%CI: −18.0 to −3.54], *p* = 0.005); age, sex and NT-proBNP levels (−8.0% [95%CI: −15.1 to −0.87], *p* = 0.029), and eGFR (−10.4% [95%CI: −17.2 to −3.49], *p* = 0.004).

**Figure 6:**
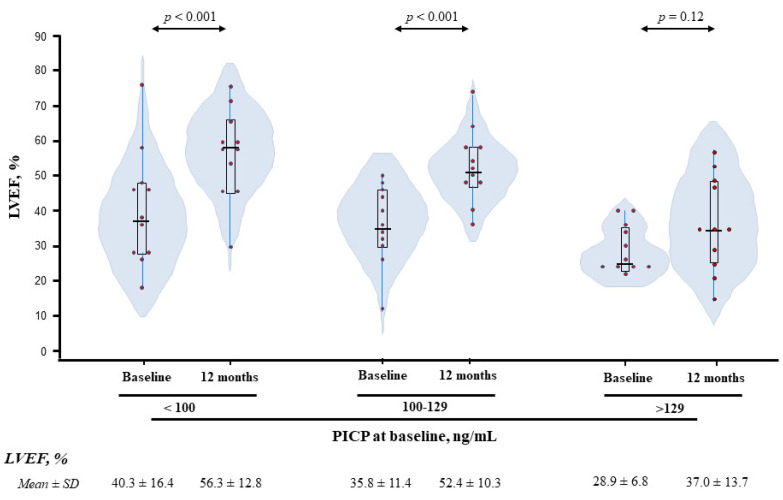
PICP levels at baseline and 1-year changes in LVEF in ACC-HF patients. Violin plots depict LVEF at baseline and after 12 months of follow-up in ACC-HF patients categorized according to tertiles of baseline PICP. Red dots are individual values, horizontal lines indicate medians and boxes indicate interquartile ranges. PICP, procollagen type-I C-terminal propeptide; LVEF, left ventricular ejection fraction; ACC-HF, anthracycline-based cancer chemotherapy-induced heart failure.

LVEF was also measured after 12 months of guideline-guided treatment in 33 ACC-HF patients. These patients were categorized according to baseline PICP tertiles in patients with low (1st tertile: <100 ng/mL, *n* = 11), medium (2nd tertile: 100–129 ng/mL, *n* = 11) and high (3rd tertile: >129 ng/mL, *n* = 11) PICP levels. As shown in Table 7, patients in the highest PICP tertile had lower HTA prevalence, were more frequently treated with mineralocorticoid receptor antagonists and digoxin, and exhibited lower LVEF. Baseline LVEF-adjusted analyses revealed that, after one year, LVEF improved in patients with low or medium PICP but remained unchanged in those with high PICP levels at baseline (Figure 6). Further adjustment by hypertension and by treatment with MRA and digoxin rendered similar results (difference in LVEF at 1-year versus baseline: 1st tertile, *p* < 0.001; 2nd tertile, *p* < 0.001; 3rd tertile = 0.051).

## 4. Discussion

The major findings of this study are the following: (1) In BC patients, serum NT-proBNP and hs-TnT increased after ACC therapy completion and serum PICP increased at 3 months post-ACC compared with baseline; (2) this increment in PICP was more pronounced in patients who developed early subclinical LVD compared with the remaining patients, and was directly and independently associated with a relative reduction of GLS; (3) the early increase in PICP predicted cardiotoxicity 1 year after completion of treatment, adding prognostic value to the early assessment of GLS and LVEF; (4) in ACC-HF patients, high levels of PICP at baseline were associated with impaired LV contractility, and with lack of cardiac functional improvement after 12 months. To our knowledge, this is the first study evaluating the pro-fibrotic effects of ACC chemotherapy, as assessed non-invasively by using circulating biomarkers associated with histologically-proven myocardial interstitial fibrosis.

It has been widely demonstrated that NT-proBNP and hs-TnT levels increase during cancer therapy, although their utility for cardiotoxicity prediction has been questioned [6,13,17,18,19,20,21,22]. In this study, we have reported ACC-induced increments in these biomarkers associated with an early reduction of LV contractility, as assessed by GLS at 3 months after ACC completion, with NT-proBNP also showing an association with a higher risk of cardiotoxicity 12 months after ACC completion. In addition, we have observed in BC patients that ACC-induced early increments in NT-proBNP and hs-TnT levels are followed by an increase in PICP levels, a biomarker associated with collagen deposition in the myocardium [9,10]. This increment in PICP is particularly evident in patients with early signs of subclinical LVD. Interestingly, these results agree with previous observations correlating changes in GLS with focal and diffuse fibrosis [23]. Moreover, a pronounced increase in PICP early after the completion of ACC therapy is associated with the development of LVEF-assessed cardiotoxicity 1 year after treatment, adding prognostic value to the assessment of GLS and LVEF early changes. Importantly, these observations have been obtained in two independent cohorts of BC patients. Finally, an association between PICP and decreased LV contractility in the context of ACC-HF was observed. Even more, longitudinal pilot observations suggest that PICP levels at baseline may influence the evolution of LV function over time in ACC-HF patients.

These findings reinforce the notion that not only cardiomyocyte injury but also the activation of fibrotic processes is relevant for the impairment of LV contractile function in cancer patients treated with ACC [7]. Specifically, our results suggest that the ACC-induced cardiomyocyte damage is followed by a fibrotic response that, if excessive, may worsen cardiac function. In this regard, we cannot discard other processes besides direct cardiomyocyte damage (i.e., ACC-induced fibroblast activation and/or inflammation) that may induce the fibrotic response observed in these patients. Mechanistically, these effects combined may result in a larger accumulation of collagen encircling cardiac muscle with reduced functionality, further restricting the stretching of muscle fibers and impairing the direct cell-to-cell communication necessary for synchronous contraction of cardiomyocytes [8]. In this regard, several studies demonstrate a detrimental effect of myocardial fibrosis on the deformation of the LV wall (i.e., reduced GLS), in experimental models [24], and in patients with different cardiomyopathies [25,26,27,28]. Of notice, the PICP levels here observed in BC patients with early subclinical LVD and later cardiotoxicity, and in patients with ACC-HF, were similar to those found in hypertensive HF patients with severe myocardial interstitial fibrosis [29]. It is also important to acknowledge the potential influence of a systemic reparative response after ACC treatment with activation of fibrotic processes in organs other than the heart, which could have an impact on the levels of serum PICP.

Altogether, these data suggest that the assessment of PICP serum levels early after ACC therapy may help to stratify cancer patients according to the risk of developing cardiotoxicity. Those at higher risk could benefit from additional anti-fibrotic therapies to improve their prognosis. In this regard, there is growing evidence suggesting that the myocardial collagen matrix is a dynamic entity and that collagen can change in content and composition in response to therapy [8]. For instance, treatment with mineralocorticoid receptor antagonists has been reported to reduce serum levels of PICP in patients at risk of HF [30] and in patients with HF [31,32]. In this context, whether monitoring cardioprotective therapies in ACC-treated cancer with cardiotoxicity may benefit from serial PICP determinations during treatment remains to be investigated.

Some limitations of the present study must be acknowledged. First, the small sample size may have constrained statistical power to detect differences in the different biomarkers assessed in this study, particularly in ACC-HF patients, and therefore preclude from adequately assessing the impact of potential confounders in the association and longitudinal analyses. Second, cancer patients undergoing ACC treatment may suffer from volume depletion (nausea, emesis) or weight loss that may confound echocardiographic findings. Third, there is a lack of follow-up information beyond the considered treatment period in BC patients, but long-term follow-up is planned and ongoing. Fourth, serum samples after 1-year follow-up were not available to measure PICP in ACC-HF patients. Fifth, our data cannot be extrapolated to oncologic patients treated with other anti-cancer drugs different from those evaluated in this study. Sixth, because they are descriptive, the associations found between PICP and subclinical LVD or cardiotoxicity do not establish causality. Finally, we cannot discard extra-cardiac sources of PICP. In this regard, determining the extracellular volume fraction in the myocardium using cardiac magnetic resonance could have enriched the description of the influence of myocardial fibrosis on PICP serum levels in ACC-treated patients.

## 5. Conclusions

This study shows for the first time that, in women with BC, increments in the biomarkers reflecting cardiomyocyte injury and hemodynamic stress, hs-TnT and NT-proBNP, in response to ACC therapy are followed by an early elevation of the circulating biomarker of myocardial collagen type I deposition, PICP. The increase in this biomarker of fibrosis is particularly pronounced in those patients with signs of early subclinical LVD. Importantly, a large increment of PICP predicts the future development of ACC-induced LVD with more robustness than NT-proBNP. On the contrary, no prognostic value has been observed for hs-TnT in the cohorts of BC women here evaluated. We have also confirmed the association of PICP and NT-proBNP with LVD in pilot observations obtained from patients with ACC-HF. In addition, high levels of the biomarker of fibrosis were associated with a lack of improvement in LV contractility over time. These results provide additional evidence that, in addition to cardiomyocyte stress, myocardial fibrosis is a cardiotoxic side-effect of ACC treatment contributing to LVD in BC patients. Nonetheless, these preliminary findings should be considered as a hypothesis-generating study, supporting the need for further studies designed to evaluate the prognostic potential of PICP, either by itself or combined with other biomarkers of myocardial remodeling, to monitor cardiotoxicity in larger cohorts of patients under chemotherapy.

## Figures and Tables

**Table 1:** Baseline clinical characteristics in all BC patients and in patients categorized according to the absence or presence of subclinical left ventricular dysfunction (LVD) at 3 months after completion of ACC therapy (CUN cohort).

Baseline Clinical Characteristics	All Patients (*n* = 87)	Subclinical LVD		*p*-Value
		No (*n* = 69)	Yes (*n* = 18)	
Age, years	50.8 ± 11.6	50.7 ± 11.7	51.1 ± 11.7	0.90
BMI, kg/m^2^	23.9 ± 4.5	23.8 ± 4.5	24.1 ± 4.2	0.81
Oncologic parameters, *n* (%)				
Breast cancer side				
Left	48 (55.2)	37 (53.6)	11 (61.1)	
Right	38 (43.7)	31 (44.9)	7 (38.9)	0.77
Bilateral	1 (1.1)	1 (1.4)	0 (0.0)	
Ki67, %	40.6 ± 24.0	41.0 ± 25.3	39.1 ± 19.1	0.73
HER2 positive, *n* (%)	21 (24.1)	17 (24.6)	4 (22.2)	0.83
TNM Stage, *n* (%)				
I	16 (18.4)	14 (20.3)	2 (11.1)	
II	44 (50.6)	31 (44.9)	13 (72.2)	
III	23 (26.4)	21 (30.4)	2 (11.1)	0.19
IV	4 (4.6)	3 (4.3)	1 (5.6)	
Epirubicin, mg/m^2^	384 (353–396)	390 (362–398)	361 (274–392)	0.06
Taxanes, *n* (%)				
Paclitaxel	27 (31.0)	23 (33.3)	4 (22.2)	0.36
Docetaxel	60 (69.0)	46 (66.7)	14 (77.8)	
Other treatments, *n* (%)				
Carboplatin	20 (23.0)	16 (23.2)	4 (22.2)	0.93
Anti-HER2				
Trastuzumab	10 (11.5)	8 (11.6)	2 (11.1)	0.97
Trastuzumab + Pertuzumab	11 (12.6)	9 (13.0)	2 (11.1)	
Surgery before ACC, *n* (%)	27 (31.0)	25 (36.2)	2 (11.1)	0.040
Radiotherapy, *n* (%)	13 (14.9)	12 (17.4)	1 (5.6)	0.21
Cardiovascular comorbidities, *n* (%)				
Hypertension	14 (16.1)	11 (15.9)	3 (16.7)	0.94
Obesity	10 (11.5)	8 (11.6)	2 (11.1)	0.95
Dyslipidemia	17 (19.5)	14 (20.3)	3 (16.7)	0.73
Diabetes Mellitus	2 (2.3)	1 (1.4)	1 (5.6)	0.37
Current smoking	7 (8.0)	6 (8.7)	1 (5.6)	0.66
eGFR < 60 mL/min/1.73 m^2^	2 (2.3)	2 (2.9)	0 (0.0)	
Atrial Fibrillation	1 (1.1)	1 (1.4)	0 (0.0)	
Cardiovascular treatments, *n* (%)				
ACE inhibitor	4 (4.6)	3 (4.3)	1 (5.6)	0.83
Angiotensin receptor blocker	5 (5.7)	3 (4.3)	2 (11.1)	0.28
Beta-blockers	2 (2.3)	1 (1.4)	1 (5.6)	0.37
Diuretics	2 (2.3)	1 (1.4)	1 (5.6)	0.37
Lipid-lowering drug	9 (10.3)	8 (11.6)	1 (5.6)	0.68
Glucose-lowering drug	2 (2.3)	1 (1.4)	1 (5.6)	0.37
Liver function parameters				
AST, IU/L	18.0 (15.0–20.0)	18.0 (15.0–20.0)	15.0 (13.0–18.5)	0.05
ALT, IU/L	13.0 (11.0–18.0)	14.0 (12.0–18.0)	12.5 (9.3–16.8)	0.18
GGT, IU/L	17.0 (13.0–26.0)	18.0 (13.0–28.0)	15.0 (12.0–18.3)	0.13
ALP, IU/L	60.0 (50.0–72.0)	61.0 (49.0–76.0)	59.5 (48.8–69.3)	0.35
Echocardiographic parameters				
GLS, %	−20.6 ± 2.0	−20.5 ± 2.0	−21.2 ± 2.0	0.19
LVEF (3D), %	63.8 ± 5.3	63.9 ± 5.4	63.4 ± 5.0	0.70
Biomarkers				
Cardiomyocyte stress/damage				
NTproBNP, pg/mL	41.4 (24.2-64.2)	41.1 (23.8–63.3)	46.5 (28.2–71.8)	0.34
hs-TnT, ng/L	3.0 (3.0-4.5)	3.0 (3.0–4.2)	3.0 (3.0–4.9)	0.87
Myocardial fibrosis				
PICP, ng/mL	72.0 (59.5-86.5)	74.4 (61.0–88.1)	65.7 (57.6–77.9)	0.26
CITP:MMP1 ratio	1.5 (1.2-3.0)	1.6 (1.2–3.2)	1.5 (1.0–2.5)	0.17

BC means breast cancer; ACC, anthracycline-based cancer chemotherapy; BMI, body mass index; TNM, Tumor, Node, Metastasis; HER2, human epidermal growth factor receptor 2; eGFR, estimated glomerular filtration rate; ACE, angiotensin-converting enzyme; AST, aspartate aminotransferase; ALT, alanine aminotransferase; GGT, gamma-glutamyltransferase; ALP, alkaline phosphatase; GLS, global longitudinal strain; 3D, three dimensional; LVEF, left ventricular ejection fraction; NT-proBNP, amino-terminal pro-brain natriuretic peptide; hs-TnT, high sensitivity troponin T; PICP, procollagen type I C-terminal propeptide; CITP, collagen type I C-terminal telopeptide; MMP-1, matrix metalloproteinase-1. Quantitative variables are expressed as mean±SD or median (interquartile range) and categorical variables as number (percentage).

**Table 2:** Linear mixed regression analyses for the association with global longitudinal strain in BC patients 3 months after completion of ACC therapy (CUN cohort).

Clinical Characteristics	Univariable		Multivariable	
	Estimate (95%CI)	*p*-Value	Estimate (95%CI)	*p*-Value
Age, years	0.02 (−0.01 to 0.04)	0.09	−0.02 (−0.05 to 0.02)	0.34
BMI, kg/m^2^	0.04 (−0.02 to 0.10)	0.15	0.03 (−0.03 to 0.09)	0.34
Left breast, yes/no	−0.20 (−0.69 to 0.28)	0.41		
Ki67, %	0.002 (−0.01 to 0.01)	0.76		
Epirubicin cumulative dose, mg/m^2^	0.005 (0.001 to 0.01)	0.018	−0.004 (−0.008 to 0.001)	0.15
Paclitaxel, yes/no	−0.22 (−0.74 to 0.30)	0.40		
Other treatments, yes/no				
Carboplatin	0.19 (−0.38 to 0.76)	0.50		
Anti-HER2	−0.01 (−0.56 to 0.54)	0.98		
Surgery before ACC	0.03 (−0.51 to 0.56)	0.92		
Radiotherapy	0.09 (−0.60 to 0.78)	0.79		
Comorbidities, yes/no				
Hypertension	0.19 (−0.50 to 0.89)	0.58		
Obesity	0.05 (−0.73 to 0.84)	0.89		
Dyslipidemia	0.24 (−0.39 to 0.87)	0.46		
Current smoking	−0.41 (−1.29 to 0.47)	0.36		
Renal function parameter				
eGFR, mL/min/1.73 m^2^	−0.02 (−0.03 to−0.01)	0.006	−0.01 (−0.03 to 0.01)	0.32
Liver function parameters (log2)				
AST, IU/L	0.35 (−0.09 to 0.80)	0.12	0.17 (−0.26 to 0.60)	0.44
ALT, IU/L	0.16 (−0.10 to 0.42)	0.23		
GGT, IU/L	−0.03 (−0.25 to 0.19)	0.78		
ALP, IU/L	0.27 (−0.20 to 0.74)	0.27		
Biomarkers (log2)				
NT-proBNP, pg/mL	0.29 (0.10 to 0.47)	0.003	0.26 (0.06 to 0.46)	0.010
hs-TnT, ng/L	0.25 (0.01 to 0.49)	0.043	0.10 (−0.17 to 0.36)	0.48
PICP, ng/mL	0.94 (0.51 to 1.37)	<0.001	0.88 (0.45 to 1.31)	<0.001
CITP:MMP-1 ratio	0.05 (−0.16 to 0.27)	0.62		
GLS at baseline, %			0.80 (0.68 to 0.93)	<0.001

Abbreviations as in Table 1.

**Table 3:** Baseline clinical characteristics of BC patients categorized according to the absence or presence of cardiotoxicity at 12 months after completion of ACC therapy.

Baseline Clinical Characteristics	CUN Cohort			HULAFE Cohort		
	Cardiotoxicity		*p*-Value	Cardiotoxicity		*p*-Value
	No (*n* = 55)	Yes (*n* = 10)		No (*n* = 59)	Yes (*n* = 11)	
Age, years (min-max)	50.1 ± 11.6	51.3 ± 12.5	0.76	56.1 ± 12.8	46.8 ± 10.1	0.026
BMI, kg/m^2^	23.2 ± 3.7	24.8 ± 5.0	0.24	26.5 ± 5.0	24.6 ± 4.6	0.26
TNM Stage, *n* (%)						
I	12 (21.8)	2 (20.0)		10 (17.0)	4 (36.4)	
II	26 (47.3)	5 (50.0)	0.89	36 (61.0)	4 (36.4)	0.24
III	14 (25.5)	3 (30.0)		13 (22.0)	3 (27.3)	
IV	3 (5.5)	0 (0.0)				
Oncologic treatment, *n* (%)						
CUN cohort						
Epirubicin, mg/m^2^	384 (354–397)	375 (272–392)	0.58			
Taxanes, *n* (%)						
Docetaxel	39 (70.9)	10 (100)	0.06			
Paclitaxel	16 (29.1)	0 (0.0)				
HULAFE cohort						
ACC1				15 (25.4)	7 (63.6)	0.012
ACC2				44 (74.6)	4 (36.4)	
Current smoking, *n* (%)	3 (5.5)	1 (10.0)	0.58	9 (15.3)	1 (9.1)	0.59
eGFR < 60 mL/min/1.73 m^2^, *n* (%)	1 (1.8)	0 (0.0)		1 (1.7)	0 (0.0)	
Cardiovascular comorbidities, *n* (%)						
Hypertension	7 (12.7)	2 (20.0)	0.54	17 (28.8)	1 (9.1)	0.17
Obesity	4 (7.3)	2 (20.0)	0.20	12 (20.3)	1 (9.1)	0.38
Dyslipidemia	7 (12.7)	2 (20.0)	0.54	13 (22.0)	3 (27.3)	0.70
Diabetes Mellitus	1 (1.8)	0 (0.0)		5 (8.5)	2 (18.2)	0.32
Cardiovascular treatment, *n* (%)						
ACE inhibitor	1 (1.8)	1 (10.0)	0.29	3 (5.1)	2 (18.2)	0.17
Angiotensin receptor blocker	3 (5.5)	1 (10.0)	0.50	9 (15.3)	1 (9.1)	0.59
Beta-blockers	1 (1.8)	0 (0.0)		5 (8.5)	2 (18.2)	0.32
Diuretics	0 (0.0)	0 (0.0)		8 (13.6)	1 (9.1)	0.68
Lipid-lowering drug	5 (9.1)	2 (20.0)	0.31	8 (13.6)	3 (27.3)	0.25
Glucose-lowering drugs	1 (1.8)	0 (0.0)		5 (8.5)	2 (18.2)	0.32
Echocardiographic parameters						
GLS, %	−20.7 ± 1.9	−20.1 ± 2.3	0.34	−16.1 ± 1.9	−17.5 ± 1.8	0.056
LVEF (2D), %				66.5 ± 5.9	68.8 ± 5.6	0.24
LVEF (3D), %	63.6 ± 4.8	61.7 ± 5.8	0.28			
Biomarkers						
NT-proBNP, pg/mL	38.7 (24.2–62.1)	52.7 (9.8–90.4)	0.98	60.8 (29.1–118)	48.0 (33.6–98.7)	0.81
hs-TnT, ng/L	3.0 (3.0–4.6)	3.0 (3.0–4.1)	0.49	7.2 (3.0–10.7)	3.0 (3.0–7.6)	0.14
PICP, ng/mL	66.6 (58.2–87.5)	69.1(58.8–89.0)	0.93	75.9 (59.1–107)	60.7 (53.0–80.5)	0.07

ACC, protocol treatment with anthracyclines as explained in methods. The remaining abbreviations as in Table 1. Quantitative variables are expressed as mean±SD or median (interquartile range) and categorical variables as number (percentage).

**Table 4:** Differences in echocardiographic parameters and biomarkers after completion of ACC therapy (post-ACC), 3 months after ACC (3m-post-ACC), and 12 months after ACC (12m-post-ACC) in BC patients with presence versus those with absence of cardiotoxicity at 12m-post-ACC.

Parameters	Difference vs Absence of Cardiotoxicity									*p* for Interaction
	Post-ACC			3m-Post-ACC			12m-Post-ACC			
	Difference	95% CI	*p*-Value	Difference	95% CI	*p*-Value	Difference	95% CI	*p*-Value	
CUN cohort										
Echocardiographic parameters										
GLS, %	0.14	−0.87 to 1.15	0.79	0.69	−0.62 to 2.01	0.30	1.53	−0.22 to 3.28	0.09	0.40
LVEF (3D), %	−0.25	−3.31 to 2.82	0.87	-1.83	−5.03 to 1.37	0.26	−11.2	−15.0 to −7.38	<0.001	<0.001
Biomarkers (log2)										
Cardiomyocyte stress/damage										
NTproBNP, pg/mL	0.30	−0.49 to 1.08	0.46	0.31	−0.43 to 1.06	0.41	−0.92	−1.94 to 0.09	0.08	0.41
hs-TnT, ng/L	0.25	−0.16 to 0.66	0.24	-0.17	−0.63 to 0.29	0.47	−0.19	−0.72 to 0.33	0.47	0.35
Myocardial fibrosis										
PICP, ng/mL	0.06	−0.15 to 0.27	0.57	0.44	0.20 to 0.68	<0.001 *	0.10	−0.19 to 0.40	0.50	0.014 *
HULAFE cohort										
Echocardiographic parameters										
GLS, %	−1.17	−2.56 to 0.21	0.10	1.28	−0.21 to 2.77	0.09	4.08	1.45 to 6.70	0.002	<0.001
LVEF (2D), %	−1.06	−5.00 to 2.88	0.60	−2.34	−5.83 to 1.15	0.19	−11.8	−14.6 to −9.06	<0.001	<0.001
Biomarkers (log2)										
Cardiomyocyte stress/damage										
NTproBNP, pg/mL				0.65	0.03-1.28	0.041	0.68	−0.07-1.42	0.08	0.45
hs-TnT, ng/L				−0.03	−0.68 to 0.61	0.92	0.14	−0.30 to 0.57	0.54	0.51
Myocardial fibrosis										
PICP, ng/mL				0.74	0.47 to 1.01	<0.001 *	0.13	−0.20 to 0.46	0.43	<0.001 *

Abbreviations as in Table 1. * Significant after Benjamini and Hochberg multiple test correction (5% FDR) in the biomarker analyses.

**Table 5:** Partly conditional logistic models to analyze longitudinal associations between repeated assessments of changes in biomarkers from baseline to 3m-post-ACC and risk of cardiotoxicity at 12m-post-ACC.

Biomarkers	CUN Cohort			HULAFE Cohort		
	OR	95% CI	*p*-value	OR	95% CI	*p*-Value
**PICP**						
Model 1	3.42	1.40 to 8.60	0.007 *	7.94	2.46 to 30.6	0.001 *
Model 2	3.44	1.38 to 8.76	0.008 *	9.67	2.39 to 46.1	0.002 *
Model 3	3.49	1.40 to 8.90	0.008 *	12.8	3.09 to 68.3	0.001
Model 4	2.95	1.13 to 7.64	0.025 *	8.13	2.03 to 41.4	0.006 *
**Hs-TnT**						
Model 1	0.78	0.42 to 1.40	0.40	0.44	0.18 to 0.96	0.050
**NT-proBNP**						
Model 1	1.48	1.04 to 2.18	0.038	1.72	0.76 to 4.72	0.24
Model 2	1.48	1.01 to 2.26	0.052			
Model 3	1.44	1.00 to 2.14	0.06			
Model 4	1.14	0.79 to 1.71	0.50			

Odds ratios (OR) are expressed for a doubling in biomarker levels. The remaining abbreviations as in Table 1. Model 1: adjustment by baseline biomarker value; Model 2: adjustment by age, ACC treatment, and baseline biomarker value; Model 3: adjustment by age, cardiovascular comorbidities, and baseline biomarker value; Model 4: adjustment by eGFR, and baseline biomarker value. * Significant after Benjamini and Hochberg multiple test correction (5% FDR).

**Table 6:** Prediction improvement evaluation after adding PICP to models including GLS and LVEF changes from baseline to 3m-post-ACC.

Echocardiographic Parameters	CUN Cohort			HULAFE Cohort		
	Estimate	95% CI	*p*-Value	Estimate	95% CI	*p*-Value
**GLS**						
Discrimination improvement						
ROC analyses						
AUC_GLS_	0.600	0.4860.715	0.08	0.754	0.6450.862	<0.001
AUC_GLS + PICP_	0.679	0.5680.789	0.002	0.804	0.7070.901	<0.001
ΔAUC	0.079	0.0080.149	0.030	0.050	0.0190.080	0.002
Reclassification improvement						
IDI	0.072	0.020–0.125	0.007	0.067	0.022–0.112	0.004
Continuous NRI						
Events	0.121	−0.220–0.453	0.49	0.364	0.018–0.646	0.023
Non-events	0.259	0.123–0.453	<0.001	0.220	0.104–0.347	<0.001
All	0.380	0.022–0.735	0.036	0.584	0.242–0.886	<0.001
LVEF *						
Discrimination improvement						
ROC analyses						
AUC_LVEF_	0.624	0.514–0.734	0.028	0.530	0.402–0.659	0.64
AUC_LVEF + PICP_	0.683	0.579–0.787	<0.001	0.663	0.535–0.793	0.013
ΔAUC	0.059	0.006–0.112	0.029	0.133	0.008–0.259	0.037
Reclassification improvement						
IDI	0.077	0.025–0.129	0.003	0.121	0.044–0.198	0.002
Continuous NRI						
Events	0.152	−0.159–0.472	0.35	0.182	−0.250–0.579	0.40
Non-events	0.346	0.203–0.482	<0.001	0.229	0.042–0.396	0.011
All	0.497	0.148–0.849	0.005	0.411	−0.043–0.831	0.065

ROC means receiving operating characteristic; AUC, area under the curve; IDI, integrated discrimination index; NRI, net reclassification index. The remaining abbreviations as in Table 1. * Three-dimensional (3D) in the CUN cohort, two-dimensional (2D) in the HULAFE cohort.

**Table 7:** Baseline clinical characteristics of ACC-HF patients with echocardiography at 1-year follow-up classified according to PICP tertiles.

Baseline Clinical Characteristics	PICP Tertiles, ng/mL			*p* for Trend
	1st (*n* = 11)	2nd (*n* = 11)	3rd (*n* = 11)	
PICP, ng/mL	<100	100–129	>129	
Age, years	61.5 ± 12.6	59.4 ± 11.5	60.6 ± 11.1	0.87
Female gender, *n* (%)	10 (90.9)	10 (90.9)	7 (63.6)	0.10
BMI, kg/m^2^	28.9 ± 6.1	26.7 ± 4.8	28.3 ± 5.0	0.80
Sodium, mmol/L	139 ± 2.4	139 ± 2.4	138 ± 3.3	0.13
Potassium, mmol/L	4.1 ± 0.4	4.1 ± 0.7	4.2 ± 0.5	0.61
Hemoglobin, g/dL	12.5 ± 0.9	12.6 ± 1.9	12.6 ± 1.8	0.89
eGFR, mL/min/1.73 m^2^	69.2 ± 26.4	73.0 ± 36.8	59.9 ± 30.8	0.49
NYHA Class, *n* (%)				
III-IV	3 (27.3)	1 (9.1)	6 (54.5)	0.17
HF duration, months	2.0 (1.0–24.0)	1.0 (1.0–4.0)	8.0 (1.0–84.0)	0.20
≥2 previous hospitalizations, *n* (%)	1 (9.1)	1 (9.1)	1 (9.1)	>0.99
Comorbidities, *n* (%)				
Ischemic heart disease	0 (0.0)	0 (0.0)	1 (9.1)	
Diabetes mellitus	3 (27.3)	3 (27.3)	4 (36.4)	0.65
Hypertension	8 (72.7)	2 (18.2)	2 (18.2)	0.009
Atrial fibrillation	0 (0.0)	0 (0.0)	1 (9.1)	
Hypercholesterolemia	3 (27.3)	5 (45.5)	5 (45.5)	0.39
Treatments, *n* (%)				
ACEIs/ARBs	9 (81.8)	11 (100)	10 (90.9)	0.47
Beta-Blockers	11 (100)	9 (81.8)	11 (100)	>0.99
Diuretics	7 (63.6)	8 (72.7)	10 (90.9)	0.14
MRA	6 (54.5)	6 (54.5)	11 (100)	0.022
Digoxin	1 (9.1)	1 (9.1)	6 (54.5)	0.014
Statins	7 (63.6)	5 (45.5)	7 (63.6)	0.80
LVEF, %	40.3 ± 16.4	35.8 ± 11.4	28.9 ± 6.8	0.034
NT-proBNP, pg/mL	1563 (396–2167)	1510 (346–4990)	1726 (960–7300)	0.08

ACC-HF, anthracycline-based chemotherapy-induced heart failure; BMI, body mass index; eGFR, estimated glomerular filtration rate; NYHA, New York Heart Association; ACEI, angiotensin-converting enzyme inhibitor; ARB, angiotensin II type 1 receptor blockers; MRA, mineralocorticoid receptor antagonist; LVEF, LV ejection fraction; NT-proBNP, N-terminal pro–B-type natriuretic peptide; PICP, procollagen type I C-terminal propeptide. Quantitative variables are expressed as mean ± SD or as median (interquartile range). Categorical variables are expressed as numbers (percentages).

## Data Availability

The data presented in this study are available on request from the corresponding author.

## Supplementary Materials

The following supporting information can be downloaded at: https://www.mdpi.com/article/10.3390/cancers14122941/s1, Table S1: Clinical characteristics in BC patients at baseline, after completion of ACC therapy (post-ACC), and 3 months after ACC (3m-post-ACC) (CUN cohort); Table S2: Differences in echocardiographic parameters and in biomarkers after completion of ACC therapy (post-ACC) and 3 months after ACC (3m-post-ACC) in BC patients with presence versus those with absence of subclinical left ventricular dysfunction (LVD) at 3 months after completion of ACC therapy (CUN cohort); Table S3: Echocardiographic parameters and biomarkers in BC patients at baseline, after completion of ACC therapy (post-ACC), 3 months post-ACC (3m-post-ACC) and 12 months post-ACC (12m-post-ACC); Table S4: Baseline clinical characteristics of BC patients categorized according to the absence or presence of cardiotoxicity at 12 months after completion of ACC therapy (CUN cohort).
