# Supplementary material for: A Fibrosis Biomarker Early Predicts Cardiotoxicity Due to Anthracycline-Based Breast Cancer Chemotherapy
Source: Cancers (Basel). 2022 Jun 14;14(12):2941. doi: 10.3390/cancers14122941 (PMC9221256; doi:10.3390/cancers14122941)
Supplement: Supplementary file 1 [file cancers-14-02941-s001.zip › cancers-1723528-supplementary.pdf]

cancers

MDPI

Supplementary Materials

A fibrosis biomarker early predicts cardiotoxicity due to anthracycline-based breast cancer chemotherapy

Ana de la Fuente, Marta Santisteban, Josep Lupón, José Manuel Aramendía, Agnes Díaz, Ana Santaballa, Amparo Hernándiz, Pilar Sepúlveda, Germán Cediel, Begoña López, José María López Picazo, Manuel M. Mazo, Gregorio Rábago, Juan José Gavira, Ignacio García-Bolao, Javier Díez, Arantxa González, Antoni Bayés-Genís and Susana Ravassa

Table S1. Clinical characteristics in BC patients at baseline, after completion of ACC therapy (post-ACC), and 3 months after ACC (3m-post-ACC) (CUN cohort).

	**Baseline**	**Post-ACC**	**3m-post-ACC**
Renal function parameter			
eGFR, mL/min/1.73m2	95.3 ± 16.2	103 ± 16.8*	101 ± 18.7*
Liver function parameters			
AST, IU/L	18.0 (15.0-20.0)	19.0 (15.0-25.0)*	20.0 (17.0-25.0)*
ALT, IU/L	13.0 (11.0-18.0)	20.0 (15.0-36.0)*	18.0 (14.0-27.5)*†
GGT, IU/L	17.0 (13.0-26.0)	24.0 (17.0-35.0)*	19.0 (14.0-28.0)†
ALP, IU/L	60.0 (50.0-72.0)	67.0 (55.0-78.0)*	63.0 (51.0-76.0)†
Echocardiographic parameters			
GLS, %	-20.6±2.0	-20.5±2.4	-19.6±2.5*†
LVEF (3D), %	63.8±5.3	63.3±4.5	62.3±5.9
Biomarkers‡			
Cardiomyocyte stress/damage			
NTproBNP, pg/mL	41.4 (24.4-64.1)	71.5 (42.9-117)*	57.1 (35.2-96.2)*
hs-TnT, ng/L	3.0 (3.0-4.5)	8.1 (4.0-11.1)*	8.0 (4.9-11.9)*
Myocardial fibrosis			
PICP, ng/mL	72.0 (59.5-86.5)	61.8 (51.1-74.0)	87.6 (69.2-119)*†
CITP:MMP-1 ratio	1.5 (1.2-3.0)	2.1 (1.2-3.6)	2.0 (1.3-3.8)

BC means breast cancer; ACC, anthracycline-based cancer chemotherapy; eGFR, estimated glomerular filtration rate; AST, aspartate aminotransferase; ALT, alanine aminotransferase; GGT, gamma glutamyltransferase; ALP, alkaline phosphatase; 3D, three-dimensional; LVEF, left ventricular ejection fraction; NT-proBNP, amino-terminal pro-brain natriuretic peptide; hs-TnT, high sensitivity troponin T; PICP, procollagen type I C-terminal propeptide; CITP, collagen type I C-terminal telopeptide; MMP-1, matrix metalloproteinase-1. Quantitative variables are expressed as mean±SD or as median (IQR) and categorical variables as number (percentage). *P<0.05 vs basal, †P<0.05 vs post-ACC. ‡P values in the biomarker analyses were adjusted according to the Benjamini and Hochberg multiple-test correction (5% FDR)

Table S2. Differences in echocardiographic parameters and in biomarkers after completion of ACC therapy (post-ACC) and 3 months after ACC in BC patients with presence versus those with absence of subclinical left ventricular dysfunction (LVD) at 3 months after ACC (CUN cohort).

	Difference vs absence of subclinical LVD						P for interaction
	Post-ACC			3m-post-ACC			
	Difference	95% CI	P value	Difference	95% CI	P value	
Echocardiographic parameters							
GLS, %	0.82	0.09 to 1.55	0.028	4.34	3.45 to 5.24	<0.001	<0.001
LVEF (3D), %	0.57	-1.61 to 2.76	0.61	-5.53	-8.68 to -2.38	0.001	<0.001
Biomarkers (log2)							
Cardiomyocyte stress/damage							
NTproBNP, pg/mL	0.63	0.19 to 1.07	0.005	0.51	-0.05 to 1.07	0.08	0.25
hs-TnT, ng/L	-0.03	-0.59 to 0.53	0.91	0.08	-0.34 to 0.51	0.70	0.89
Myocardial fibrosis							
PICP, ng/mL	-0.01	-0.21 to 0.20	0.93	0.70	0.46 to 0.95	<0.001*	<0.001*
CITP:MMP-1 ratio	-0.09	-0.57 to 0.38	0.71	0.05	-0.53 to 0.63	0.88	0.60

Abbreviations as in table S1. *Significant after Benjamini and Hochberg multiple-test correction (5% FDR) in the biomarker analyses

Table S3. Echocardiographic parameters and biomarkers in BC patients at baseline, after completion of ACC therapy (post-ACC), 3 months post-ACC (3m-post-ACC) and 12 months post-ACC (12m-post-ACC).

	Baseline	Post-ACC	3m-post-ACC	12m-post-ACC
**CUN cohort**				
Echocardiographic parameters				
GLS, %	-20.6±2.0	-20.7±2.4	-19.7±2.1*†	-19.5±2.8*†
LVEF (3D), %	63.3±4.9	63.4±4.4	62.7±5.4	61.5±7.3
Biomarkers&				
Cardiomyocyte stress/damage				
NTproBNP, pg/mL	39.0 (24.1-64.2)	68.4 (42.9-117)*	54.9 (34.2-95.5)*	46.0 (21.0-78.5)
hs-TnT, ng/L	3.0 (3.0-4.5)	8.6 (4.0-12.0)*	7.5 (4.3-10.0)*	4.6 (3.0-6.4)*†‡
Myocardial fibrosis				
PICP, ng/mL	66.6 (59.0-87.5)	64.6 (51.9-74.3)	85.1 (69.8-115)*†	90.7 (70.8-117)*†
**HULAFE cohort**				
Echocardiographic parameters				
GLS, %	-16.5±1.9	-15.5±2.5*	-15.2±2.0*	-15.7±2.6
LVEF (2D), %	66.9±5.8	65.0±5.8	62.5±5.5*	61.6±6.3*
Biomarkers&				
Cardiomyocyte stress/damage				
NT-proBNP, pg/mL	60.6 (29.5-99.5)		74.2 (40.7-120)*	94.1 (55.0-157)*†
hs-TnT, ng/L	6.9 (3.0-10.6)		12.6 (8.9-19.2)*	9.3 (7.1-11.4)*†
Myocardial fibrosis				
PICP, ng/mL	73.3 (57.8-99.4)		87.7 (66.8-113)*	93.3 (80.4-123)*

Abbreviations as in table S1. Quantitative variables are expressed as mean±SD or as median (IQR) and categorical variables as number (percentage) *P<0.05 vs basal, †P<0.05 vs post-ACC, ‡P<0.05 vs 3m-post-ACC. &P values in the biomarker analyses were adjusted according to the Benjamini and Hochberg multiple-test correction (5% FDR).

Table S4. Baseline clinical characteristics of BC patients categorized according to the absence or presence of cardiotoxicity at 12 months after completion of ACC therapy (CUN cohort).

	**Cardiotoxicity**		**P value**
	**No (n=55)**	**Yes (n=10)**	
Oncologic parameters, n (%)			
Breast cancer side			
Left	31 (56.4)	6 (60.0)	
Right	23 (41.8)	4 (40.0)	0.90
Bilateral	1 (1.8)	0 (0.0)	
Ki67, %	40.7 ± 25.2	42.5 ± 27.4	0.84
HER2 positive, n (%)	14 (25.5)	3 (30.0)	0.76
Other treatments, n(%)			
Carboplatin	17 (30.9)	2 (20.0)	0.49
Anti-HER2			
Trastuzumab	9 (16.4)	0 (0.0)	0.10
Trastuzumab+Pertuzumab	5 (9.1)	3 (30.0)	
Radiotherapy, n(%)	46 (83.6)	9 (90.0)	0.61
Surgery before ACC, n (%)	18 (32.7)	2 (20.0)	0.71
Liver function parameters			
AST, IU/L	17.5 (14.0-20.0)	17.5 (13.8-20.3)	0.69
ALT, IU/L	13.0 (11.5-18.5)	12.0 (9.5-14.0)	0.78
GGT, IU/L	17.0 (13.0-27.0)	17.0 (14.8-42.3)	0.33
ALP, IU/L	61.0 (51.0-75.0)	53.0 (38.5-66.0)	0.10
Biomarkers			
CITP:MMP1 ratio	1.5 (1.2-3.3)	2.0 (1.2-3.2)	0.85

Abbreviations as in table S1. Quantitative variables are expressed as mean±SD or median (interquartile range) and categorical variables as number (percentage).
